# A Narrative Review on Sleep and Eating Behavior

**DOI:** 10.1007/s11892-025-01611-4

**Published:** 2025-09-30

**Authors:** Wing Yee Cheng, Wai Sze Chan

**Affiliations:** Department of Psychology, The University of Hong Kong, Pokfulam, Hong Kong

**Keywords:** Narrative review, Sleep duration, Sleep timing, Sleep quality, Food consumption, Dietary habits, Type 2 diabetes

## Abstract

**Purpose of Review:**

This narrative review synthesizes current evidence on the role of various sleep parameters—including sleep duration, sleep quality, sleep timing, social jetlag, and chronotype—in energy intake, macronutrient consumption, diet quality, and meal timing. We aim to evaluate whether existing evidence supports a causal impact of sleep on eating behavior and discuss the clinical implications of these findings for diabetes care and management.

**Recent Findings:**

The impact of short sleep duration on eating behavior is the most widely studied and supported by experimental evidence suggesting that reduced sleep duration increases energy intake and promotes poorer diet quality. Later sleep timing is also associated with increased energy intake and poorer diet quality, and may interact with short sleep duration in influencing eating behavior. Chronotype, social jetlag, and sleep quality have also been linked to eating behavior; however, findings in these areas have been predominantly observational and cross-sectional, and may be confounded by co-occurring influences from other sleep parameters.

**Summary:**

Given the strength of the evidence for the role of sleep duration in eating behavior, future studies should evaluate the feasibility and efficacy of sleep extension interventions for controlling energy intake and improving diet quality in patients with type 2 diabetes. Further research should also clarify and distinguish the independent and interacting influences of multiple sleep parameters on eating behavior, as well as the potential effects of eating behavior on sleep.

## Introduction

Sleep is a well-recognized factor that influences various health conditions including physical and mental health [1, 2]. A number of sleep parameters including sleep duration, sleep timing, and sleep quality, have been shown to be determinants of obesity and obesity-related risks [3] and glycemic control [4–6]. Diabesity, a term describing diabetes occurring in the context of obesity [7], is influenced by sleep through various mechanisms [8]. Although the causal mechanisms underlying the relationships between sleep and various diabesity-related factors require further investigations [9], the impact of sleep on eating behavior is likely one of the contributors to sleep’s influence on diabesity-related outcomes [10, 11].

Several systematic reviews have evaluated the impact of sleep parameters, such as sleep duration [10, 12, 13], sleep quality [11], sleep timing [14], social jetlag [15], and chronotype [16], on eating behavior [10–17]. This narrative review aims to provide an updated overview of the current evidence for the impact of these sleep parameters on several aspects of eating behavior, namely, energy intake, macronutrient consumption, diet quality, defined as the adequacy and balance of nutrients [18], and meal timing, referring to the time and regularity of meals and snacks. This review will focus on the most recent and robust findings and their implications on diabetes care and management. It should be noted that emerging evidence suggests a bidirectional relationship between sleep and eating behavior, with eating behavior also influencing sleep. However, this review focuses only on the impact of sleep on eating behavior.

We identified relevant literature using Google Scholar searches. Search strings included sleep-related terms (e.g., sleep, sleep deprivation, sleep restriction, sleep extension, sleep quality, sleep timing, chronotype, circadian rhythm) and terms related to eating behavior (e.g., eating behavior, energy intake, food intake, food choice, diet quality, meal composition). We prioritized articles published within the previous 5 years at the time of searching the literature. When recent evidence was limited, earlier research studies were included in the review. We screened the titles and abstracts for relevance, following by reviewing the full text of potentially eligible articles. We focused on peer-reviewed journal publications and did not consider other publications such as dissertations or conference abstracts. Evidence was synthesized narratively and thus no meta-analysis was undertaken.

## Sleep Duration

Sleep duration is the most widely studied sleep parameter in relation to eating behavior. The National Sleep Foundation has specified the recommended sleep duration for each age group. Adults are recommended to sleep 7 to 9 h per 24-hour cycle [19]. Short sleep duration, often operationalized as sleeping less than 6 h, has been found to be associated with increased energy intake, increased eating occasions (especially during the evening), poor diet quality, and irregular meal timing in both observational and experimental studies. For instance, in several population-based studies in the USA, Sweden, and China, adults who were habitual short sleepers reported greater energy intake, greater consumption of caffeine and sugar, more snacking after dinner, and more irregular meal timing than those who had adequate sleep [20–24]. The associations between short sleep duration and poorer diet quality and irregular meal timing have also been observed in adults with cardiovascular diseases, metabolic syndrome, and type 2 diabetes [25, 26]. Although these associations have been well-established in observational studies, they may not represent causal effects of short sleep duration on eating behavior.

Experimental sleep restriction studies have been conducted to evaluate the causal impact of short sleep duration on eating behavior, primarily in healthy populations. A meta-analysis of the effect of experimentally induced short sleep duration on energy intake has shown that partial sleep deprivation (≤ 5.5 h per night) led to increased energy intake by 204 calories on average per day [12]. Significant variabilities in the effect sizes were observed across studies, with larger effects in experiments involving 5 or more nights of partial sleep deprivation whereas single-night total sleep deprivation did not lead to increased energy intake [12], suggesting that the effect of sleep loss on eating may occur only when a certain threshold of sleep loss is cumulated. Some of the variabilities across studies may also be attributed to the variabilities in the methods used to assess eating behaviors. For instance, eating was measured using single meal observations in the laboratory in some studies (15-minute breakfast buffet [27]; 30-minute afternoon tea buffet [28]), but multiple meals occurring in free-living environments were measured in others (9 to 12 meals over 3 days [29]; 3 meals over an 18-hour period [30]).

The variabilities in the effects of partial sleep deprivation on eating behavior may also be explained by the time at which eating behavior was assessed. Nedeltcheva et al. found that healthy adults who were restricted to 5.5 h of sleep per night for 14 nights had greater energy intake from snacks later in the day (19:00 to 07:00) than when they were allowed 8.5 h of sleep, but the total energy intake throughout the day was not significantly different between the two conditions [31]. Similarly, Spaeth et al. found that healthy adult participants in the 4-hour sleep restriction condition for 4 nights had greater energy intake due to increased consumption of fat during late-night hours (22:00–03:59) than their counterparts in the 10-hour sleep control condition [32]. Brondel et al. found that, after 1 night of 4-hour partial sleep deprivation, healthy adult men had greater energy intake in the latter half of the day (afternoon and evening), but not the earlier half of the day (breakfast and lunch), although the difference could also be attributable to the settings; eating in the latter half of the day was assessed in free-living conditions whereas eating in the earlier half of the day was assessed in the laboratory [33]. A similar finding has also been observed in adolescents. Heathy adolescents who were restricted to 6.5 h of sleep per night for 5 nights had greater total energy intake and greater energy intake from fat and carbohydrates than when they were allowed to sleep 9.5 h per night, also only during late evening hours (21:00–01:00) [34]. These findings suggest that partial sleep deprivation may have an impact on energy intake especially later in the day. Nonetheless, the majority of prior sleep restriction experiments did not assess the timing of food consumption [35–37], preventing a definitive conclusion to be drawn. Future studies are recommended to assess the timing of food consumption and evaluate whether eating behavior later in the day is more vulnerable to the effects of partial sleep deprivation.

The most promising evidence for the impact of sleep duration on eating comes from intervention studies in which the extension of sleep duration in habitual short sleepers led to reduced energy intake [38, 39]. In these studies, participants received a single session of sleep hygiene counseling with the goal of extending time in bed by 1–1.5 h or up to 8.5 h per night. In Al Khatib et al. [38], participants in the sleep extension condition extended their sleep duration by 21 min per night for 4 weeks, and they consumed 9.6 g less free sugar per day compared to their counterparts in the habitual short sleep condition. Similarly, in Tasali et al. [39], participants in the sleep extension condition extended their sleep duration by 1.2 h per night for 2 weeks, and they consumed 270 kcal less per day on average than their counterparts in the habitual short sleep control condition. These findings provided empirical support not only for the role of sleep duration on eating behavior, but also the feasibility and clinical utility of sleep extension interventions for improving diet quality and reducing the risk of obesity. Future research shall begin to explore the feasibility, sustainability, and efficacy of sleep extension interventions for improving diet quality and reducing excess energy consumption in populations who may most benefit from such changes, such as people with obesity and type 2 diabetes.

## Sleep Timing

In addition to sleep duration, sleep timing has also been linked to poor diet quality and late-night eating. In a nationally representative sample of adolescents and adults in the United States, self-reported later bedtime was associated with lower consumption of fruits and vegetables and higher consumption of unhealthy foods and sugar-sweetened beverages even after controlling for sleep duration [40]. Similar findings were also observed when actigraphy was used to assess sleep timing in Baron et al. [41], in which individuals identified as late sleepers by actigraphy (midpoint of sleep > 05:30) reported lower consumption of fruits and vegetables and greater energy intake after 20:00 after controlling for sleep duration. Although later bedtime was associated with shorter sleep duration in Grummon et al. [40] and Baron et al. [41], its independent association with eating behavior suggests that sleep timing has a unique influence on eating behavior over and beyond the effect of sleep duration.

Not only does sleep timing matter, but it also seems to interact with sleep duration in influencing eating behavior. McNeil et al. [42] restricted healthy participants to 4 h of sleep for 1 night by either delaying bedtime or advancing wake time to the midpoint of the participant’s habitual sleep period. Following delayed bedtime for 1 night, participants consumed more carbohydrates throughout the day than they did following 7 to 8 h of sleep in the control condition, whereas no changes in macronutrient consumption were observed following advanced wake time compared to the control, despite that participants had equal amount of reduced sleep duration in both sleep restriction conditions [42]. Maloney et al. [43] extended this paradigm to 4 consecutive nights of 6-hour partial sleep deprivation with a 2-hour delay or advancement of bedtime and wake time, respectively. They found that participants in the delayed bedtime condition consumed more calories, fat, and sodium during these 4 days compared to the control condition, whereas no changes were observed in the advanced wake time condition [43]. These findings suggest that reducing sleep duration by delaying bedtime could lead to increased energy intake, especially from carbohydrates and fat, but reducing sleep duration by advancing wake time does not. These findings appear to corroborate with the other finding that increased energy intake during sleep restriction is primarily attributed to increased eating during late evening hours. Nonetheless, it remains unclear whether delaying bedtime alone without sleep restriction would produce similar effects. Moreover, as most of these studies were conducted in healthy individuals, the relevance for clinical populations requires further investigation.

## Chronotype and Social Jetlag

Circadian preference and its alignment with social and occupational scheduling have also been studied in relation to eating behavior. Chronotype reflects individual differences in circadian preference and is typically categorized as “morning”, “intermediate”, or “evening” chronotypes, referring to preferences for earlier, neutral, and later rise time and bedtime [44]. Adults who reported having an evening chronotype had later meal timing (e.g., about 2.5-hour later for breakfast; 20-minute later for lunch, and 30-minute later for dinner in [45]) and greater energy intake during dinner (e.g., + 4.7% in [46]) than people having a morning chronotype. Using actigraphy to verify chronotype, Yoshizaki and Togo [47] found that young adults with a later chronotype had less consumption of eggs and grains and more consumption of sugar than those with an earlier chronotype. This association between later chronotype and poorer dietary quality has also been observed in patients with type 2 diabetes. Those who have an evening chronotype were more likely to skip breakfast and had greater energy intake from dinner than from other meals [48].

Although people may have circadian preferences, many cannot follow their desired schedules due to work and social obligations. The misalignment between circadian preferences and social and work scheduling can lead to social jetlag, referring to the experience of having different sleep schedules on work days and non-work days with at least an hour of difference in the midpoint of sleep [49]. Individuals with larger social jetlag typically go to bed later and sleep longer on non-work days than on workdays [50]. The findings regarding the relationship between social jetlag and eating behavior have been mixed depending on the population studied. Rusu et al.’s review of sleep timing variability and eating behavior suggests that there were no significant differences in eating behavior between groups with and without social jetlag in studies conducted in healthy adults; however, greater energy intake and poorer dietary quality were found in the social jetlag group in studies conducted in populations with obesity and type 2 diabetes [14]. Positive findings were also observed in a sample of adolescents in the USA such that social jetlag was associated with lower odds of consuming breakfast and fruits and vegetables but higher odds of consuming fast-food and sweetened beverages [50]. The same pattern was also observed in a longitudinal study in post-bariatric surgery patients, in which patients with larger social jetlag consistently reported greater intakes of calories, carbohydrates, and fat across 3 timepoints spanning 6 months [51]. In a sample of university students in Japan, larger social jetlag was associated with a lower daily energy intake but a higher consumption of sugar, sweets, and chocolate [47].

The mixed findings on social jetlag and eating behavior suggest large individual differences in the effect of social jetlag on eating behavior. Moreover, they could also be attributable to the potential interactions between social jetlag and other sleep parameters such as chronotype. For instance, individuals with a morning chronotype and large social jetlag (> 2 h difference) had poorer diet quality compared to their counterparts with small social jetlag (< 1 h difference), but no differences were found in individuals with evening preference regardless of social jetlag [49]. Furthermore, the existing findings have been primarily observational and could be explained by underlying general tendencies for unhealthy lifestyle habits, such as personality traits characterized by poor impulse control and self-regulation. For instance, people who are more impulsive or who have suboptimal self-regulation abilities may be more prone to engaging in unhealthy habits, including being less adherent to a consistent bedtime as well as healthy diets, contributing to the association between the two. Future investigations are needed to evaluate the causal and potentially interactive relationships of social jetlag, chronotype, and other sleep parameters with eating behavior.

## Sleep Quality

Sleep quality is commonly assessed using self-reported questionnaires such as the Pittsburgh Sleep Quality Index (PSQI [52]) or single-item question rating sleep quality from “very poor” to “very good” [53] in previous research. Sleep quality and sleep duration are correlated but distinct concepts [54], and they have been found to be independently associated with eating behavior [23].

Poor sleep quality has been found to be associated with poor diet quality and disordered eating behavior in various populations. Poorer sleep quality was associated with uncontrolled eating, emotional eating, and night eating behavior in patients with type 2 diabetes [55]. Similar patterns were observed in community samples of adults, in which poor sleep quality was associated with emotional eating [56] and breakfast skipping, irregular mealtimes, and late-night snacking [56, 57]. Even with adequate sleep duration (7–8 h), poor sleep quality was also found to be associated with consuming more soft drinks and fast food in place of fruits, vegetables, and milk in a sample of adolescents living in South Korea [58]. Adults with poor sleep were also found to have a higher percentage of energy intake from carbohydrates (+ 1%) and a lower percentage from protein (− 1.5%) compared to good sleepers [59]. A recent cross-sectional study revealed that short sleepers with poor sleep quality had significantly lower adherence to a healthy diet, but this finding was not found among short sleepers with good sleep quality [23].

Although previous findings have shown that poor sleep quality is associated with eating behavior, these associations have been primarily observational. In addition to the possibility that poor sleep quality is a concomitant with eating behavior, these associations could also be explained by the potential effects of eating behavior on sleep quality. Increasing research has been conducted to examine the potential impact of eating behavior on sleep quality and other sleep parameters [60, 61]. Although the findings have been inconclusive, this potential direction of effect needs to be considered to fully understand the relationship between sleep quality and eating behavior.

## Discussion

In recent research, the impact of short sleep duration on eating behavior is the most widely studied and supported by experimental evidence showing that reduced sleep duration increases energy intake and promotes poorer diet quality. In experimental sleep restriction studies in which the timing of food consumption was assessed, increased energy intake occurred mainly in the latter part of the day. Future studies are needed to further evaluate if eating behavior later in the day is more vulnerable to the effects of sleep duration and the underlying mechanisms. Later sleep timing has also been linked to poorer diet quality and increased energy intake, and it may interact with short sleep duration in influencing eating behavior. Evidence supports the distinct impacts of sleep duration and sleep timing on eating behavior; however, further investigations, especially those that carefully distinguish the independent and interacting influences of sleep duration and sleep timing on eating behavior are needed. For instance, well-powered randomized controlled trials consisting of a combination of restricted sleep duration, normal sleep duration, delayed bedtime, and advanced bedtime conditions would be required to dissociate the causal and potentially interacting impacts of sleep duration and sleep timing on eating behavior.

Given the strength of the evidence for the role of sleep duration in eating behavior, future studies evaluating the efficacy of sleep extension interventions for improving diet quality and reducing night-time eating should be encouraged, especially in populations that may particularly benefit from such interventions. For instance, consuming meals early in the day rather than in the evening promotes better postprandial glycaemia [62]. Interventions that promote adequate sleep duration and earlier bedtime may facilitate earlier meal timing and improve diabetes outcomes in patients with type 2 diabetes.

Chronotype and social jetlag have also been linked to eating behavior; however, the findings have been predominantly observational and cross-sectional, and they may be explained by underlying general tendencies for unhealthy lifestyle habits. Poor sleep quality has also been linked to poor diet quality and greater energy intake; however, measures of sleep quality are confounded by other sleep parameters (e.g., sleep duration). Future research is needed to clarify the independent contributions of chronotype, social jetlag, and sleep quality to eating behavior. Moreover, the associations between these sleep parameters and eating behavior may greatly depend on sample characteristics. Extending the study of healthy participants to other populations, especially populations most affected by poor eating behavior such as patients with diabesity, would be beneficial.

Finally, the measurement of eating behavior remains a critical methodological issue in the research on eating behavior and energy intake. While some questionnaire measures of eating behavior have been validated, actual eating behavior are known to be difficult to measure and may differ significantly from self-report data [63]. Laboratory-based measurements are more accurate but may have limited ecological validity. Future utilization of digital and mobile technologies to improve the accuracy of the measurement of eating behavior in real-life is warranted.

## Limitations

This review did not aim to be exhaustive, and hence, may not have reviewed all available evidence. It also did not review all sleep parameters such as sleep disorders. Rather, this review provided an update of the evidence in this area of research and highlighted the most robust evidence for the impact of sleep duration and delayed bedtime on increased energy intake and poorer diet quality. The current evidence supports the clinical utility and feasibility of sleep extension interventions for improving glycemic control in patients with type 2 diabetes.

This narrative review has several methodological limitations. Unlike systematic reviews or meta-analyses, this review did not employ a systematic search strategy, potentially introducing selection biases. Moreover, this review did not conduct formal quality assessments of the included studies and did not present quantitative synthesis of the evidence. These methodological constraints limit the strength of the conclusion. Furthermore, as this review focused on the impact of sleep on eating behavior, it did not evaluate the existing evidence on the bidirectional relationship between sleep parameters and eating behavior. Previous studies reported that meal composition (e.g., glycemic index and Mediterranean diet pattern) and chrononutrition factors (e.g., meal timing and frequency) can influence sleep quality and sleep duration [64–66]. Future reviews on the relationship between sleep and eating behavior will benefit from considering the evidence for the impact of eating behavior on sleep.

## Conclusion

To conclude, the existing literature suggests that partial sleep deprivation can increase energy intake and promote poorer diet quality. Sleep extension interventions have shown promising results in improving diet quality and reducing energy intake. Sleep extension can be an integral part of a comprehensive lifestyle intervention for diabetes care and management. Poor sleep quality, later sleep timing, later chronotype, and social jetlag have also been linked to greater energy intake, poorer diet quality, and irregular meal timing; however, the majority of the evidence is correlational and observational. Future research is needed to further evaluate the role of these sleep parameters on eating behavior, especially the potential interacting impact between these sleep parameters and sleep duration on eating behavior.

## Data Availability

No datasets were generated or analysed during the current study.
